# Bioinspired tumor-homing nanoplatform for co-delivery of paclitaxel and siRNA-E7 to HPV-related cervical malignancies for synergistic therapy

**DOI:** 10.7150/thno.41228

**Published:** 2020-02-10

**Authors:** Cong Xu, Wan Liu, Yuan Hu, Weiping Li, Wen Di

**Affiliations:** 1 Department of Obstetrics and Gynecology, Renji Hospital, School of Medicine, Shanghai Jiao Tong University, Shanghai 200127, People's Republic of China.; 2 Shanghai Key Laboratory of Gynecologic Oncology, Renji Hospital, School of Medicine, Shanghai Jiao Tong University, Shanghai 200127, People's Republic of China.

**Keywords:** cancer cell membrane camouflage, homotypic targeting, paclitaxel, siRNA-E7, cervical cancer

## Abstract

Because of the complexity of cancer, a combination of chemotherapy and gene therapy is an emerging treatment modality. To realize the full potential of this strategy, a smart, highly biocompatible nanosystem that enables the precise co-delivery of small-molecule anticancer drugs and small interfering RNA (siRNA) is urgently needed. This study aimed to improve the therapeutic effect against cervical cancer by using cancer cell membrane-camouflaged nanoparticles for simultaneous delivery of paclitaxel (PTX) and siRNA targeting E7.

**Methods**: By camouflaging HeLa cell membranes onto siRNA/PTX co-loaded (lactic-co-glycolic acid) (PLGA) nanoparticles, a biomimetic dual-drug delivery system (Si/PNPs@HeLa) was developed to simultaneously deliver PTX and siRNA targeting E7. After evaluating the physicochemical characteristics as well as their cell uptake and biodistribution behavior, studies on the RNA interference efficiency and antitumor ability of Si/PNPs@HeLa *in vitro* and *in vivo* were further carried out.

**Results**: The Si/PNPs@HeLa was capable of delivering PTX and siRNA simultaneously to HeLa cells both *in vitro* and *in vivo*. Moreover, benefiting from the recognition and adhesion molecules on the surface of HeLa cells, Si/PNPs@HeLa exhibited an improved immune escape ability and an increased tumor region accumulation (3-fold higher than bare nanoparticles). As a result, an excellent synergistic anti-tumor effect was observed in the HeLa tumor-bearing mice, with tumor volume inhibiting rates of 83.6% and no side effects in major organs. The mechanistic studies confirmed that E7 knockdown sensitized HeLa cells to PTX chemotherapy, mainly by inhibiting PTX-induced AKT pathway activation.

**Conclusion**: Si/PNPs@HeLa, by integrating immune escape and tumor-homing ability, can serve as an efficient dual-drug delivery system to achieve precise treatment of cervical cancer through chemo-gene combined therapy.

## Introduction

Cervical cancer (CC) is a prevalent gynecological malignancy, causing over 300,000 deaths worldwide each year 1. As a front-line chemotherapeutic agent, paclitaxel (PTX) plays a pivotal role in the treatment of cervical cancer. However, the therapeutic efficacy of PTX is limited, with response rates ranging from 29% to 63% 2-4. This is mainly due to the inability of its single anti-tumor mechanism to combat the heterogeneity of the tumor microenvironment and the complexity of the signaling pathways that regulate cancer progression. Based on the in-depth understanding of the molecular mechanisms leading to tumorigenesis, the chemo-gene combined therapy, as a promising strategy to overcome chemoresistance and achieve synergistic treatment of cancer, has been extensively studied in recent years 5-7. Persistent high-risk HPV infection is the leading cause of CC. The carcinogenicity of these HPV is mainly due to the activity of the oncoproteins E6 and E7 8, 9. The latter constitutes an attractive gene therapeutic target, as its genetic conservation is considered to be critical to carcinogenesis 10. Furthermore, based on the potential of E7 in the regulation of ATP-binding cassette (ABC) transporter 11, it might be involved in affecting the response of tumor cells to chemotherapy. In fact, several previous studies have reported that E6/E7 silencing can induce apoptosis in CC cell lines 12, 13, and the combination of gene therapy targeting HPV E6/E7 with chemotherapy can enhance the anti-tumor effect in HPV-related CC 14, 15.

Since its gene silencing effect on mammalian cells was first reported in 2001^16^, small interfering RNA (siRNA) has rapidly become a promising tool for gene therapy. However, because of its poor stability in systemic circulation and low bio-membrane permeability, efficient delivery of siRNA to its application sites is one of the key challenges toward realizing its full potential 17. On the other hand, small-molecule chemotherapy drugs and much larger siRNA molecules have very different physicochemical properties, which may lead to the differences in biodistribution of the two agents 18. In order to achieve maximum synergy, suitable nanocarriers are needed to achieve temporary co-localization of drugs and siRNA in tumor cells.

Poly (lactic-co-glycolic acid) (PLGA)-based nanoparticles (NPs) have good physicochemical properties in terms of safety, protecting genetic material from degradation, and achieving sustained release 18-20, thereby providing a good platform for carrying chemotherapy drugs and siRNA simultaneously. However, their defects, such as rapid clearance by the reticuloendothelial system (RES) and lack of specific targeting, limit their use in drug delivery. Decorating NPs with polyethylene glycol (PEG) provides "stealth" brushes that mimic the cell's glycocalyx and delays immune clearance. However, potential immunogenicity and kidney damage caused by PEG have been recently reported 21. In terms of target delivery, it can be achieved, in principle, by modifying the NPs with targeting moieties. Nevertheless, these “bottom-up” approaches require complex synthetic routes and are far from adequate in mimicking a complex protein composition on the surface of nanoparticles 22. Compared to the "bottom-up" approach, cell membrane coating nanotechnology presents a novel “top-down”strategy 23-25. By camouflaging nanoparticles with cell membranes, it faithfully preserved the composition and functionality of the cell membrane, endowing nanomaterials with various unique characteristics of the source cells, such as long systematic circulation of erythrocytes 22, 26, injured tissues-homing behavior of platelets 27, virus-binding capability of certain host cell 28, and the directed migration ability of NK cells to tumor tissue 29, 30.

Cancer cells, despite its notoriety, exhibit unique properties, such as the abilities of immune escape and “self-homing”, which are mainly mediated by membrane proteins on their surface. By completely replicating these surface antigenic structures from cells to nanoparticles, cancer cell membrane coating technology endows NPs with long circulation capability and drives NPs to self-target the homologous tumor 31. In addition, because of the unlimited reproduction ability of cancer cells, it is easy to culture them in vitro and obtain their membrane material. In fact, some studies have reported its advantages in cancer imaging 32 and therapy 33-35, as well as its prospects in clinical application 36.

**Scheme 1:**
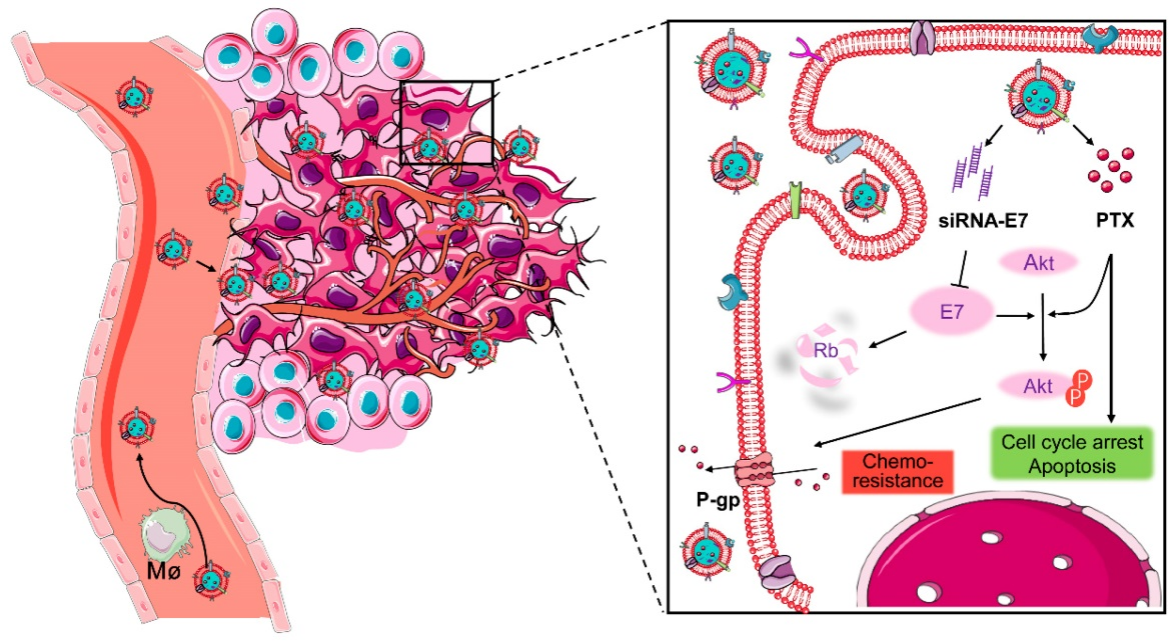
Co-delivery of paclitaxel and siRNA-E7 using a bioinspired tumor-homing nanoplatform to synergistically treat HPV-associated cervical malignancies.

Here, a bioinspired cancer cell membrane-biomimetic PLGA-based nanocarrier was developed. PTX and siRNA targeting HPV18-E7 were encapsulated in PLGA to form a core, which was subsequently covered with membranes from HeLa cancer cells to build a homologous targeting nanoparticle. Owing to the tumor-homing ability and high biocompatibility endowed by the retention of HeLa cell antigens and membrane structures on the surface of nanoparticles, Si/PNPs@HeLa showed an improved tumor preferential accumulation and cell uptake. Moreover, E7 knockdown could not only restore the expression of the anti-tumor protein Rb but also upgraded the intracellular PTX concentration by inhibiting the activation of the AKT pathway and the expression of MDR1. As a result, synergistic anticancer effects were achieved and further enhanced by the cell membrane coating technique (Scheme 1).

## Materials and Methods

### Materials

PLGA, with a lactide-glycolide ratio of 50:50 and Mw of 7,000-17,000 (ester terminated), PEI (average Mw 25,000 Da), poly (vinyl alcohol) (PVA, 30-70 kDa) and Coumarin-6 were provided by Sigma Aldrich. DiR Iodide and DiO were obtained from Yeasen. PTX was purchased from J& K Chemical Technology. The Avanti mini extruder and polycarbonate porous membrane were obtained from Avanti Polar Lipids. Antibodies targeting Rb (4H1), p-AKT (D9E), AKT (40D4), E7 (F-7) and MDR1 (D-11) were bought from Cell Signaling Technology and Santa Cruz Biotechnology, respectively. AS1411 aptamer (Ap sequence: 5′‐SH‐C6‐TTGGTGGTGGTGGTTGTGGTGGTGGTGG‐3′, 28 bp) was synthetized by Sangon Bioengineering (Shanghai, China). siRNA sequences targeting E7 (sense 5′-GCGACUCAGAGGAAGAAAATT-3′, antisense 5′-UUUUCUUCCUCUGAGUCGCTT-3′) and Cy5-labeled siRNA-NC were synthetized by Shanghai GenePharma.

The human cervical cancer cell line HeLa was obtained from the Cell Bank of the Chinese Academy of Sciences (Shanghai, China). The cells were cultured according to the guidelines recommended by the ATCC.

Female BALB/c nude mice were obtained from Shanghai Jiao Tong University School of Medicine and raised in the SPF animal facility. All the animal experimental procedures were conducted with the approval of the Animal Experimentation Ethics Committee of Jiao Tong University.

### Cancer cell membrane extraction

Cell membrane extraction was performed following the reported protocol with minor modifications 37. Briefly, HeLa cells were collected by centrifuging at 1500 rpm for 5 min, resuspended in a hypotonic lysing buffer containing PMSF, and homogenized on ice with 20 strokes in a Dounce homogenizer. The cell lysate was centrifuged at 3200 ×*g* for 10 min. All the supernatant was pooled and centrifuged at 15,000 rpm for 30 min. The resulting cell pellets were further washed with TE buffer (10 mM Tris-HCl, 1 mM EDTA) and recollected by centrifugation as before. The pellets were considered as purified HeLa cell membrane.

### Synthesis and characterization of Si/PNPs@HeLa

The double emulsion technique was applied to formulate a Si/PNPs core 18. Briefly, 10 mg PLGA, 33 µg PEI, and 0.25 mg PTX were dissolved in 0.5 mL of dichloromethane (DCM). The aqueous phase (50 µL RNase-free water containing 5 nmol siRNA) was emulsified with the above DCM solution, over an ice bath using a probe sonicator (Scientz Biotechnology, China) at 20% power and pulsing (2 s on/ 2 s off) for 5 min. 3 mL of 2% (w/v) PVA aqueous solution was added into the above primary emulsion and sonicated under the same dispersion setting to form a double emulsion. The DCM was removed by a rotary evaporator (Yarong, China) under reduced pressure to form NPs. The NPs were collected by centrifuging at 15,000 rpm for 20 min at 4 °C and washed twice with double-distilled water to remove PVA and unentrapped drugs. For DiR or Coumarin-6 labeling, 100 µg of the dye were added to the PLGA dichloromethane solution and the same procedure was followed as for the preparation of Si/PNPs.

To prepare HeLa cell membrane vesicles, the harvested purified HeLa cell membrane was extruded 11 times through a 1000 nm polycarbonate membrane (Whatman) in an Avanti mini-extruder. The resulting membrane vesicles were then coated on the drug-loaded PLGA cores by coextruding for 11 times through a 400 nm polycarbonate membrane. Dynamic light scattering (DLS; Zen 3600 Zetasizer, Malvern) and transition electron microscopy (TEM; Hitachi HT7700, Japan) were used for characterizing particle size, zeta potential, and morphology. For the stability study, nanoparticles were suspended in PBS at 37 °C, and were periodically removed for routine analysis. The average size of nanoparticles was determined by DLS.

### Encapsulation efficiency and drug loading content

The encapsulation efficiency (EE%) of siRNA was calculated based on the concentration of free siRNA in the filtrate obtained by ultrafiltration 38. The concentration of siRNA, which was labeled with Cy5, was recorded by a fluorescence spectrophotometer (Horiba, FluoroMax-4).

PTX entrapped in NPs was extracted with acetonitrile. The concentration of PTX in acetonitrile extract was detected by high performance liquid chromatography (HPLC, Agilent, 1260II) to determine the amount of PTX loaded in NPs 18.

### *In vitro* release of siRNA and PTX

Cy5-siRNA-loaded nanoparticles were suspended in PBS (pH = 7.4) at a fixed siRNA concentration of 1 nmol/mL and incubated at 37 °C under constant rotation. At different pre-determined time points, these suspensions were ultra-filtered using an ultrafiltration tube (Milipore, MWCO = 100 kDa). Then, 1 mL filtrates were collected and measured with a fluorescence spectrophotometer, and 1 mL fresh PBS was added back to each suspension.

The PTX release behavior from PNPs, Si/PNPs, and Si/PNPs@HeLa was measured in PBS (pH = 7.4) at 37 °C by the dialysis method as previously reported 39. Briefly, dialysis tubes (MWCO = 3.5 kDa) containing 1 mL of sample were immersed into 19 mL of PBS with 1 M sodium salicylate along with shaking at 100 rpm. At indicated time points, 200 µL aliquots from the flask were removed for the PTX concentration detection by HPLC and 200 µL fresh PBS containing sodium salicylate were added back.

### Immunostaining for TEM imaging

The gold nanoparticles (15 nm) were synthesized according to a published method 40. The resulting AuNPs were connected to SH‐aptamer AS1411 through the S-Au bond by the salt-aging method previously described 41. Briefly, thiolated aptamer AS1411 was treated with TCEP (50×) at pH 5.0 for 1 h to cleave the disulfide bond. These activated DNAs were mixed with AuNPs at a molar ratio of 300:1. After incubation for 1 h, 1 M NaCl solution was added to the mixed solution 6 times at 1 h intervals to reach a final NaCl concentration of 300 mM. The sample was further incubated overnight, collected by centrifugation (15,000 rpm for 30 min at 4 °C), and washed twice with 1×TAMg buffer to remove free aptamers. For immunogold staining, Si/PNPs@HeLa were incubated with AuNPs-AS1411 in 1×TAMg buffer at 37 °C for 2 h. The samples were dropped onto the grid for 5 min and washed with 10 µL distilled water. After staining with 1% uranyl acetate, the samples were imaged using a Hitachi model microscope.

### Cancer cell membrane protein characterization using SDS-PAGE

Si/PNPs@HeLa were isolated from free HeLa cell membrane materials by centrifugation at 15,000 rpm for 30 min. The resulting pure Si/PNPs@HeLa and the HeLa membranes were lysed using RIPA lysis buffer (Beyotime^TM^), respectively. The protein concentration was quantified using the BCA Protein Assay Kit (Beyotime^TM^). Protein (50 µg) was separated by 10% sodium dodecyl sulfate polyacrylamide gel electrophoresis (SDS-PAGE) and stained by Coomassie Blue Super Fast Staining Solution (BeyoBlue^TM^) for 30 min before visualization.

### *In vitro* cellular uptake

HeLa, Ect1, LO2, and RAW264.7 cells were incubated with Cy5-labeled SiNPs or SiNPs@HeLa for 3 h, rinsed, stained with DAPI, and visualized under a fluorescence microscope (Nikon, Tokyo, Japan). The quantification was performed using FACScan flow cytometry (Beckman Coulter, Brea, CA, USA).

In order to detect the co-localization of the membrane and the cores, Cy5-labeled siRNA was loaded into the PLGA core, while DiO was applied to stain the HeLa membranes. The resulting dual-labeled SiNPs@HeLa cells were incubated with HeLa cells for 1 h. Subsequently, the cells were rinsed, fixed with 4% paraformaldehyde, and stained using DAPI before imaging under a confocal laser scanning microscope (CLSM) (Leica TCS SP8).

To estimate the ability of Si/PNPs@HeLa to deliver both agents simultaneously, Cy5-labeled siRNA and Coumarin-6 were co-loaded into the nanoparticles. The next steps were the same as those in the co-localization assay described above.

### Evaluation of gene silencing by quantitative reverse transcription (qRT-PCR)

Total RNA was extracted using Trizol reagent (Takara, Japan) following the manufacturer's instructions, then reverse-transcribed into complementary DNA using the PrimeScript RT reagent kit (Takara, Japan). qRT-PCR was carried out with a TB Green^TM^ Premix Ex Taq ^TM^ II kit (Takara, Japan) according to the manufacturer's instructions. Amplifications were performed in a QuantStudio^TM^ Dx Real-Time Instrument (Thermo). The relative transcription amount of the target gene was calculated by the ∆∆Ct method, and GAPDH was used as a standardized reference gene. The prime sequences for E7 were 5′-AGCAATTAAGCGACTCAGAGGAAG-3′ (E7-forward), and 5′-GCTCAATTCTGGCTTCACACTTAC-3′ (E7 reverse).

### Cytotoxicity assay

HeLa cells were seeded at 3,000 cells/well in 96-well plates and cultured overnight. Free drugs or drug-loaded nanoparticles were added into each well at an indicated PTX concentration and a fixed siRNA concentration (100 nM). After 48 h, cell viability was detected by Cell Counting Kit-8 (Dojindo, Kumamoto, Japan) according to the manufacturer's protocol. The absorbance was measured with a microplate reader (Thermo Scientific, MA, USA) at a wavelength of 450 nm. To estimate the biocompatibility of those nanocarriers, cell viability was assessed by the same method after 72 h of incubation with the empty NPs and the empty NPs@HeLa.

### Cell apoptosis analysis

HeLa cells were treated with SiNPs, free PTX, PNPs, PNPs@HeLa, NC/PNPs@HeLa, Si/PNPs, or Si/PNPs@HeLa at a fixed PTX dose (10 nM) and siRNA dose (100 nM) for 48 h. The cells were then harvested and double-stained using an Annexin V-FITC Apoptosis Detection Kit (BD-Pharmingen) following the manufacturer's instructions. Apoptosis rates of cells were analyzed using a FACScan flow cytometer.

### Western blot

Cells were harvested and lysed in RIPA lysis buffer (Beyotime™). Protein (40 µg) was separated by SDS-PAGE and transferred to a polyvinylidene difluoride (PVDF) membrane (Millipore). After blocking in 5% BSA for 1 h at 25 °C, the membrane was incubated with primary antibodies overnight at 4 °C, followed by IRDye 800CW as the secondary antibody. Protein strips were detected using Odyssey infrared imaging system (LI-COR Biosciences).

### *In vitro* immunogenicity assay

The immunogenicity of various formulations was assessed using cytokine release assay as previously reported 30. In brief, free PTX (10 nM), free siRNA (100 nM), NPs (200 µg/mL), HeLa membrane vesicles (200 µg/mL), NPs@HeLa (200 µg/mL), Si/PNPs (equivalent PTX and siRNA load) and Si/PNPs@HeLa (equivalent PTX and siRNA load) were incubated with THP-1 cells for 24 h, then the cell supernatant was collected to measure the inflammatory cytokines (IL-1β, IL-6, TNF-α) by ELISA.

### *In vivo* imaging and biodistribution studies

The subcutaneous HeLa xenograft model was established by inoculating HeLa cells (2 × 10^6^ cells) into the right foreleg of female BALB/c nude mice (4- to 5-week-old, 18-22 g). Whole body fluorescence images were collected using an *In Vivo* Imaging System (Xenogen, USA) (Ex/Em: 748/780) at 2, 8, 24 h, and 48 h after injection of free DiR, DiR-NPs, or DiR-NPs@HeLa at a DiR concentration of 0.4 mg/kg. For the tissue distribution study, the mice were sacrificed, and tumors and major organs were collected for *ex vivo* imaging.

*In vivo* co-delivery capacity of the nanosystem was tested using Coumarin-6/Cy5-siRNA co-loaded nanoparticles. The tumor-bearing mice were received an intravenous injection of this dual-labeled NPs at 2.5 mg/kg Coumarin-6 and 2 mg/kg siRNA. After 24 h, the tumors were collected, rinsed, and prepared for the frozen sections using a cryostat microtome (Leica CM1950). The frozen slices were fixed with 4% paraformaldehyde, counterstained with DAPI and visualized under a fluorescence microscopy.

### Assessment of circulation half-life

ICR mice were randomly grouped (3 mice at each time point in each group) and injected intravenously with Coumarin-6 loaded NPs or NPs@HeLa, respectively, with a Coumarin-6 dose of 1.5 mg/kg. A small amount of blood (30 µL) from the orbital venous plexus was collected in heparinized tubes at 2 min, 30 min, 1 h, 2 h, 4 h, 8 h, and 24 h post-administration and centrifuged at 3000 rpm for 10 min. Acetonitrile (90 µL) was added to 10 µL of the resulting supernatant (plasma) to extract the fluorescence dye. The mixture was then centrifuged at 10,000 rpm for 10 min. Supernatant (90 µL) was added into the ProxiPlate-96 black microplate and quantified by fluorescence intensity measurement with a microplate reader (Thermo Scientific, MA, USA).

### *In vivo* antitumor study

The HeLa tumor-bearing mouse model was established using the method mentioned above. When the tumors reached an approximate size of 100 mm^3^, the mice were randomly divided into 5 groups (n=5) and every 2 days administered with PBS, Taxol, PNPs, Si/PNPs, or Si/PNPs@HeLa via the tail vein, at a fixed PTX dose (6 mg/kg) and siRNA dose (2 mg/kg). The tumor volume (volume = (width^2^ × length)/2) and the changes of relative body weight of the mice were monitored every 2 days. Three days after the last treatment, the mice were sacrificed after blood collection, and tumors were collected for H&E staining, immunohistochemical (IHC) analysis and TUNEL assay. To further evaluate the biosafety of PLGA-based formulations, white blood cell (WBC) count, alanine aminotransferase, and aspartate transaminase were measured using a commercial kit (Roche Diagnostic, Switzerland). Histological sections of major organs were stained with hematoxylin-eosin and observed under a microscopy.

### Statistical Analysis

The experiments were performed in triplicate. The data were analyzed with GraphPad Prism 6.0 software (La Jolla, CA, USA). Statistical evaluations of data were performed using the Student's *t*-test or one-way analysis of variance (ANOVA). All results are expressed as the mean ± standard deviation (SD). *P*-value <0.05 was considered statistically significant.

## Results and Discussion

### Preparation and characterization of Si/PNPs@HeLa

**Figure 1:**
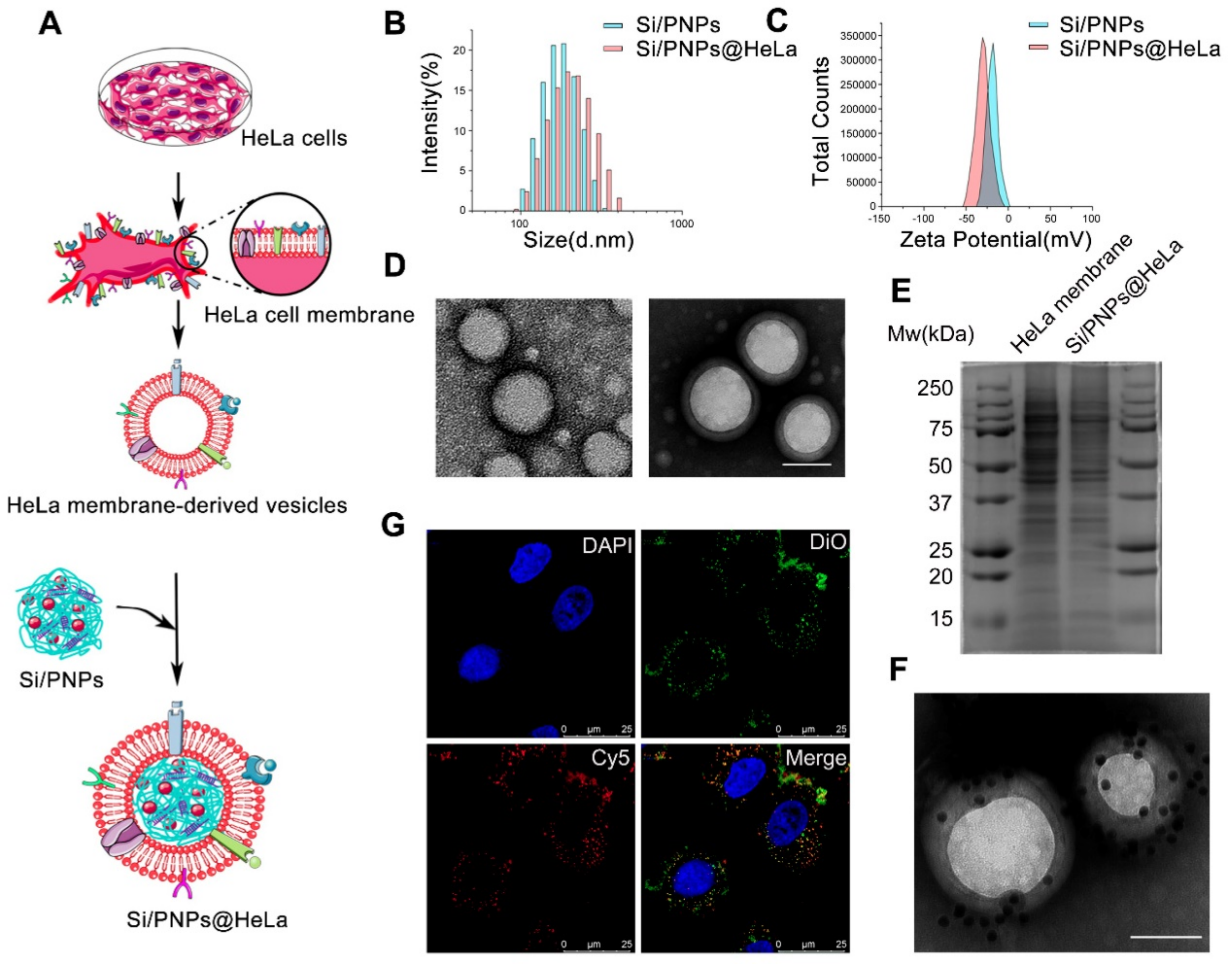
Characterization of Si/PNPs@HeLa. (A) Preparation procedure of Si/PNPs@HeLa. Size distribution (B) and ζ potential (C) of Si/PNPs (blue) and Si/PNPs@HeLa (red). (D) TEM image of Si/PNPs (left) and Si/PNPs@HeLa (right). Scale bar, 100 nm. (E) SDS-PAGE analysis of proteins in the lysate of HeLa membrane and Si/PNPs@HeLa. (F) TEM image of Si/PNPs@HeLa stained with extracellular-domain specific AuNPs-AS1411. Scale bar, 100 nm. (G) Intracellular co-localization of the HeLa membrane shell (visualized with green DiO dyes) and the SiNPs core (visualized with red Cy5-siRNA dyes).

SiRNA/PTX co-loaded nanoparticles (Si/PNPs) were formulated by a double-emulsion solvent evaporation method. A small amount of the cationic polymer, polyethyleneimine (PEI), was added during NPs formulation to increase siRNA encapsulation into NPs 42. The resulting siRNA/PTX co-loaded PLGA-polymeric cores were subsequently fused with the HeLa membrane through physical extrusion, as illustrated in Figure 1A. The control groups, PTX-loaded nanoparticles (PNPs), siRNA-loaded nanoparticles (SiNPs) and their corresponding HeLa cell membrane camouflaged nanoparticles (PNPs@HeLa and SiNPs@HeLa) were produced by the same method.

Dynamic light scattering (DLS) data showed that the size of Si/PNPs increased by about 15 nm after fusing with the HeLa membrane, and the surface charge of Si/PNPs changed from -14.4 mV to -30 mV, the latter being equivalent to that of HeLa membrane-derived vesicles, implying the success of cell membrane camouflaging (Figure 1B and C, Table S1). TEM imaging revealed that Si/PNPs@HeLa possessed a typical core-shell structure with spherical shape and good monodispersity (Figure 1D). Besides, both paclitaxel and siRNA were encapsulated efficiently in both single agent and dual agent nanoparticle formulations. The EE% of siRNA and PTX in Si/PNPs were 88.4 ± 0.22% and 90.2 ± 0.43%, respectively (Table S2).

We next performed protein gel electrophoresis followed by Coomassie blue staining to verify the successful transfer of HeLa membrane protein onto NPs, which is considered a key point to realize the biomimetic function of the membrane shell. The results showed that the protein bands of HeLa membrane lysate and Si/PNPs@HeLa lysate were identical, indicating that the cell membrane protein was well retained by the mechanical extrusion procedure (Figure 1E). Homotypic targeting has been reported to be highly dependent on the cancer cell surface interaction mediated by cellular adhesion molecules (e.g., epithelial cell adhesion molecule (EpCAM) and galectin-3). While the membrane protein CD47, acts as an anti-phagocytic receptor, is upregulated in cancer cells to mediate immune escape. Therefore, we further confirmed through western blot that these homologous targeting-related proteins and immune tolerance-related proteins have been completely transferred from cell membrane to the surface of nanoparticles (Figure S1). In addition, immunogold staining analysis of Si/PNPs@HeLa using AuNPs-AS1411, a nucleic acid aptamer-conjugated AuNP targeting the extracellular domain of nucleolin 43, 44, confirmed the correct orientation of the membrane on the Si/PNPs (Figure 1F).

To investigate the integrity of the core-shell structure during cell uptake *in vitro*, we labeled the HeLa cell membrane with DiO (green) and loaded Cy5-labeled siRNA (red) into the polymeric core. The dual-labeled nanoparticles were incubated with HeLa cells for 1 h and observed under a CLSM. The distribution of DiO and Cy5 overlapped with each other, which implied that the core-shell structure of nanoparticles remained intact for a period even after they were ingested by cells (Figure 1G).

After the structural studies, the release kinetics of siRNA and PTX were investigated *in vitro*. PTX or siRNA exhibited a sustained release profile in various formulations. Neither membrane camouflaging nor co-delivery had a notable impact on the release profile of the payload (Figure S3A and B).

### Cellular uptake

**Figure 2:**
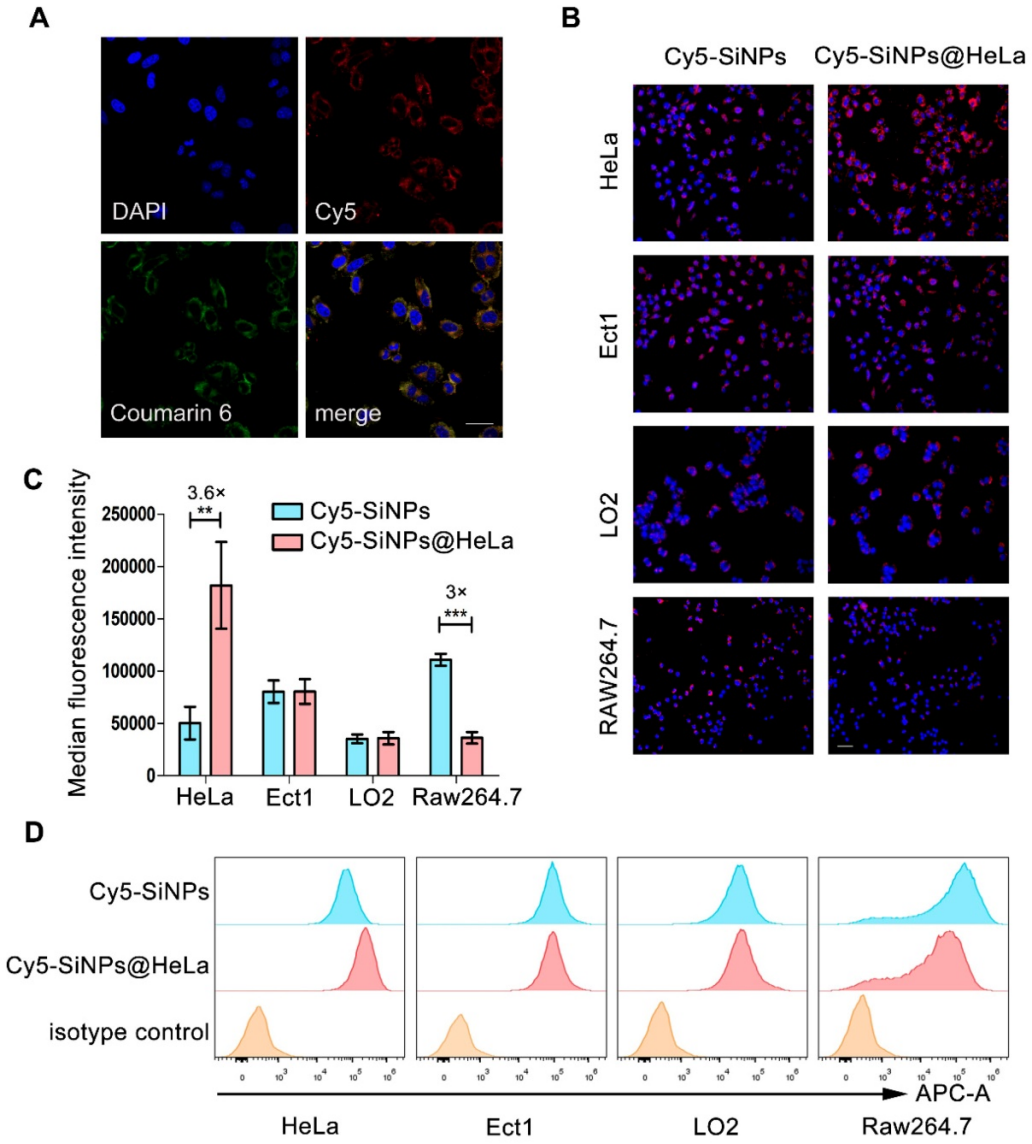
Validating co-delivery capability, homologous targeting property, and stealth ability of Si/PNPs@HeLa *in vitro*. (A) CLSM images of HeLa cells incubated with Si/PNPs@HeLa. Coumarin-6 (green), representing the location of PTX, and siRNA labeled by Cy5 (red) were co-loaded into the nanoparticles. Scale bar, 50 µm. (B) Fluorescence microscopy images of HeLa cells, Ect1 cells, LO2 cells, and RAW264.7 cells after incubation with Cy5 labeled SiNPs or SiNPs@HeLa for 3 h. Scale bar, 100 µm. The nucleus was stained with DAPI (blue); the siRNA loaded into the NPs was labeled with Cy5 (red). MFI values (C) and flow cytometric histogram (D) of each cell line treated with Cy5 labeled SiNPs or SiNPs@HeLa for 3 h. Untreated cells were used as isotype controls. Data are shown as mean ± SD (n=3). **p < 0.01, ***p <0.001.

A similar biodistribution profile of the loaded agents is essential to achieve a synergistic effect. To estimate the capability of our nanoplatform in dual-agents co-delivery, Coumarin-6 (green) was used to replace PTX and co-loaded into nanoparticles with Cy5 (red)-labeled siRNA. As shown in Figure 2A, after treatment with nanoparticles, Coumarin-6 and Cy5 were co-localized in the cytoplasm of HeLa cells.

Tumor cell membranes are considered to possess a homologous adhesion property, and this may possibly enable Si/PNPs@HeLa to exhibit a selective affinity for homologous cells. Here, Cy5 labeled siRNA was loaded into the nanoparticles, and the cellular internalization behavior of naked nanoparticles and cell membrane coating nanoparticles was investigated by incubating with several different cell lines, including HeLa cells and other non-tumor cells (e.g., immortalized human ectocervical cells (Ect1) and human normal liver cells (LO2)). As expected, compared with bare Cy5-SiNPs, Cy5-SiNPs@HeLa were more efficiently internalized by HeLa cells, which is clearly reflected by the intensity of red fluorescence in the fluorescence microscopy images (Figure 2B, Figure S4 and S5). However, little significant change in uptake rate was observed between Cy5-SiNPs and Cy5-SiNPs@HeLa when incubated with Ect1 and LO2 cells.

As foreign particles, nanoparticles are susceptible to be recognized and engulfed by macrophages once entering the body. On the other hand, tumor cells are adept at using “marker of self” on the surface to evade phagocytosis by macrophages. Hence, disguising the nanoparticles with tumor cell membranes was expected to delay macrophage-mediated clearance of nanoparticles, thereby prolonging blood circulation time and enhancing drug accumulation in tumor sites. To verify this anti-phagocytosis capability, the nanoparticles were incubated with RAW264.7 mouse macrophage cells. The results showed that the fluorescence intensity of the Cy5-SiNPs@HeLa group was obviously lower than that of the Cy5-SiNPs group (Figure 2B and Figure S5).

Furthermore, flow cytometry analysis provided quantitative evidence supporting the conclusion drawn from the fluorescence microscopy observation. Compared with Cy5-SiNPs, the median fluorescence intensity of HeLa cells treated with Cy5-SiNPs@HeLa increased by 3.6 times and that of macrophages decreased by 3 times. Meanwhile, the median fluorescence intensity was similar between Cy5-SiNPs and Cy5-SiNPs@HeLa in the other two cell lines (Figure 2C and D).

### *In vitro* antitumor effects and alteration of pathways

**Figure 3:**
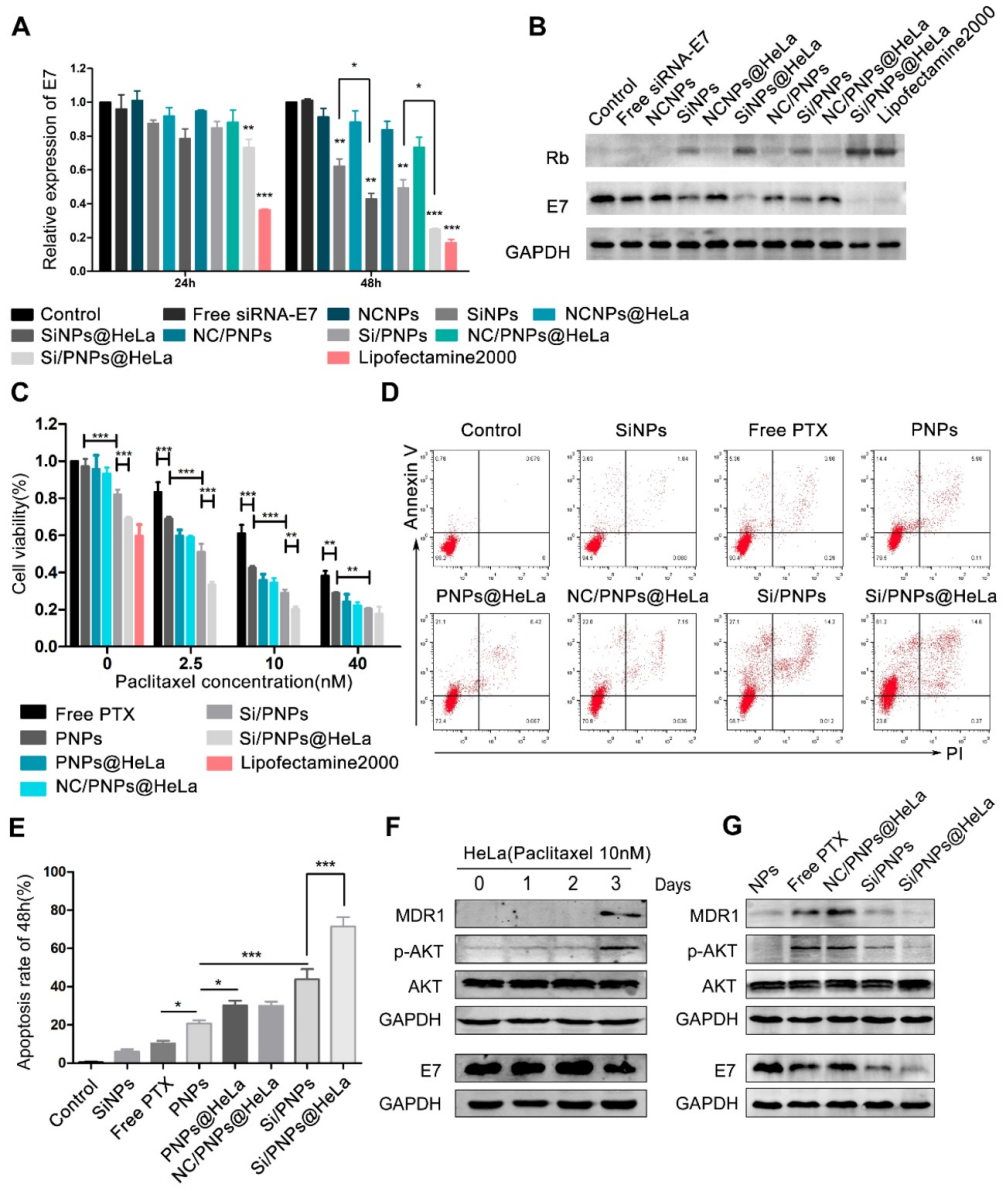
Gene silencing efficacy and cytotoxicity evaluation. Quantitative RT-PCR (A) and western blot (B) determination of E7 levels in HeLa cells treated with different siRNA-E7-based formulations at a constant siRNA concentration of 100 nM. Free siRNA-E7 and formulations containing scrambled siRNA were used as negative controls, lipofectamine 2000 was used as a positive control. (C) Cell viability of HeLa cells when treated with various formulations at indicated PTX concentrations and fixed siRNA concentrations (100 nM) for 48 h. (D) Flow cytometry analysis of HeLa cell apoptosis induced by SiNPs, free PTX, PNPs, PNPs@HeLa, NC/PNP@HeLa, Si/PNPs, and Si/PNPs@HeLa at a PTX concentration of 10 nM and an siRNA concentration of 100 nM for 48 h. (E) Corresponding statistics on the proportion of apoptotic cells. (F) Alteration of the pathways in the HeLa cells when treated for indicated times with 10 nM PTX. (G) The effects of Si/PNPs and Si/PNPs@HeLa on PTX-induced alteration of pathways in the HeLa cells, as detected by western blotting. Data are given as the mean ± SD (n=3). *p < 0.05, **p, < 0.01, ***p <0.001.

HPV oncogene E7 plays a crucial role in HPV-induced carcinogenesis and is considered to be associated with PTX resistance in cervical cancer. We therefore loaded siRNA-E7 with or without PTX in the nanoparticles and evaluated their interfering effects. According to the qRT-PCR data (Figure 3A), the knockdown effect of the HeLa membrane-coated co-loaded nanoparticles was up to 26.8 ± 5.7% and 75.2 ± 0.6%, at an siRNA dose of 100 nM after 24 and 48 h, respectively, and this was significantly higher than that of the uncoated nanoparticles. Moreover, the knockdown efficiency of Si/PNPs@HeLa at 48 h was close to that of the commercial transfection reagent Lipofectamine 2000 (83.2%). Western blotting analysis further estimated the gene silencing ability of various nanoparticles (Figure 3B). Both SiNPs@HeLa and Si/PNPs@HeLa efficiently decreased the E7 expression level in HeLa cell line and restored the expression level of its downstream protein Rb.

The dose-dependent cytotoxicity of free PTX, PNPs, PNPs@HeLa, NC/PNPs@HeLa, Si/PNPs, and Si/PNPs@HeLa was evaluated (Figure 3C). At each PTX concentration, the cell viability displayed a gradual decrease in the sequence of free PTX, PNPs, PNPs@HeLa, Si/PNPs, and Si/PNPs@HeLa. The cytotoxicity of scrambled-siRNA/PTX co-loaded nanoparticles (NC/PNPs@HeLa) was similar to that of the corresponding PTX nanoparticles (PNPs@HeLa). Compared with free PTX, PNPs and PNPs@ HeLa exhibited enhanced cell killing effects probably as a result of increased uptake. More importantly, silencing E7 expression by co-loading siRNA-E7 into the nanoparticles could sensitize HeLa cells to the PTX treatment, and this sensitizing effect could be further enhanced by membrane camouflaging. In addition, compared to the formulations containing PTX, SiNPs and SiNPs@HeLa could only partially inhibit tumor cell growth, with an inhibition rate of 18 ± 2.5% and 31 ± 1.0%, respectively. This might be achieved, as reported in previous literature, by inhibiting the cell cycle via restoring the expression of its downstream antitumor protein Rb 45.

Moreover, at a constant PTX concentration of 10 nM, we observed a gradual increase in the apoptosis rate exhibited in the following order: free PTX < PNPs < PNPs@HeLa < Si/PNPs < Si/PNPs@HeLa, as determined by PI/annexin V double staining followed by flow-cytometry analysis (Figure 3D and E). However, SiNPs-induced apoptosis was relatively low. Consistent data was observed in the Calcein-AM/PI assay (Figure S6).

It has been widely observed that increased expression of MDR1 contributes to PTX resistance in cancer cells through an efflux of chemotherapeutic agents 46, 47 and that activation of the AKT pathway is involved in MDR1-mediated drug resistance 48, 49. In line with these previous reports, our time-course experiment showed that treatment of HeLa cells with 10 nM PTX resulted in activation of the AKT pathway and an increase in the expression of MDR1 at 72 h (Figure 3F). Given the association between E7 and the AKT pathway 50, 51 and the latter in regulation of MDR1 expression, we hypothesized that E7 knockdown could sensitize the cells to PTX. Therefore, we explored the alteration of the AKT pathway and MDR1 expression in the HeLa cells after treatment for 72 h with the empty NPs, free PTX, NC/PNPs@HeLa, Si/PNPs, and Si/PNPs@HeLa. As shown in Figure 3G, free PTX and NC/PNPs@HeLa substantially induced activation of the AKT pathway and MDR1 expression in HeLa cells, and this effect was partially abolished by silencing E7 via Si/PNPs and Si/PNPs@HeLa.

### Biodistribution and pharmacokinetics

**Figure 4:**
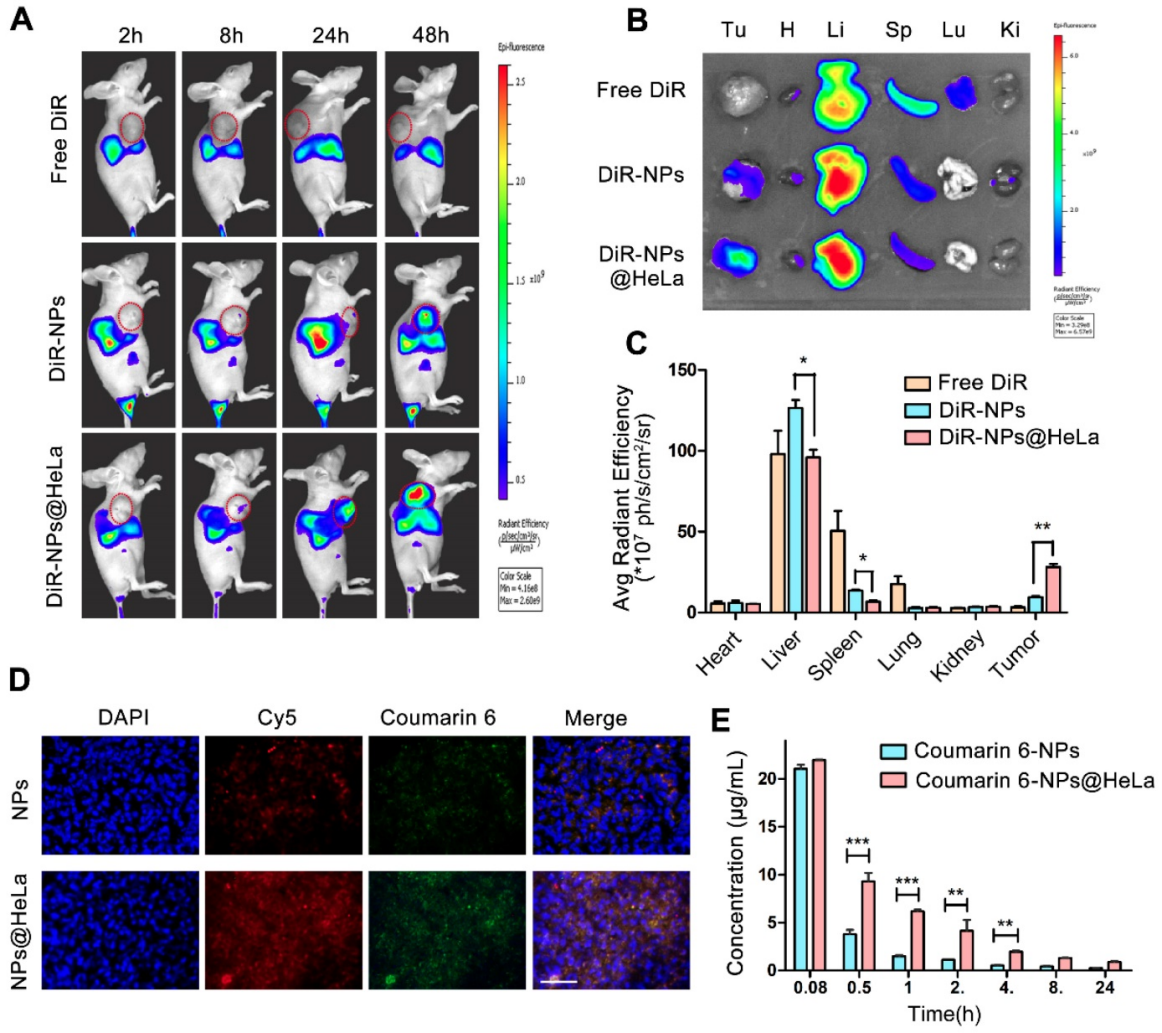
Biodistribution and pharmacokinetics of NPs and NPs@HeLa. (A) Whole body fluorescence images of HeLa subcutaneous xenograft mice at 2, 8, 24, and 48 h after injection of free DiR, DiR-NPs, and DiR-NPs@HeLa (at DiR dose of 0.4 mg/kg). The tumor area is circled in red. (B) Representative *ex vivo* images of tumor (Tu), heart (H), liver (Li), spleen (Sp), lung (Lu), and kidney (Ki) at 48 h post-injection of free DiR, DiR-NPs or DiR-NPs@HeLa. (C) The corresponding average radiation efficiency of resected tumors and major organs. (D) Representative fluorescence microscopy images of tumor cryosections 24 h after intravenous injection with dual-labeled NPs or NPs@HeLa. The nucleus was stained with DAPI (blue); Coumarin-6 (green), representing the location of PTX, and siRNA labeled by Cy5 (red) were co-loaded into the nanoparticles. Scale bar, 100 µm. (E) Blood circulation of Coumarin-6 loaded NPs and NPs@HeLa. Data are given as the mean ± SD (n = 3 mice per group). *p < 0.05, **p < 0.01, ***p <0.001.

The time-course biodistribution patterns of different formulations were examined in HeLa tumor- bearing mice by NIR-fluorescence whole body imaging. We used DiR as a tracer since the dye can be stably retained in NPs 52 and has been widely cited as a tracer for the biodistribution studies of nanoparticles 53. As shown in Figure 4A, after intravenous injection with free DiR, no marked fluorescence signal was observed in the tumor site during the observation period, while DiR-NPs achieved a small amount of tumor accumulation probably through the EPR effect. By contrast, HeLa membrane coating remarkably improved the active targeting ability of nanoparticles, as expected from the *in vitro* study, showing increased fluorescence signals in the tumors and reduced non-specific accumulation in normal organs during all the test times. Forty-eight hours after injection, *ex vivo* imaging of resected organ was performed. The quantitative estimation of biodistribution by using the average radiation efficiency showed that the tumor content of DiR-NPs@HeLa was 3-fold higher than that of DiR-NPs (Figure 4B and C), further confirming the homologous targeting ability of DiR-NPs@HeLa. On the other hand, the content of DiR-NPs@HeLa accumulated in the liver and spleen was much lower than that of DiR-NPs, and they were reduced by 24% and 50%, respectively.

To investigate whether PTX and siRNA could be simultaneously delivered to tumors, the dual- fluorophore-labeled Coumarin-6/Cy5-siRNA-loaded nanoparticles were injected intravenously into tumor-bearing mice. Twenty-four hours after injection, frozen sections of tumor tissues were taken for fluorescence analysis. Coumarin-6 (green) and Cy5 (red) overlapped at the same location (Figure 4D), indicating that co-delivery of PTX and siRNA can be achieved by this PLGA-based nanosystem. In addition, the fluorescence intensity of the tumor tissue in the NPs@HeLa group was higher than that in the NPs group, which was consistent with the data of NIR-fluorescence imaging.

We next examined the systemic circulation time of NPs and NPs@HeLa. The hydrophobic fluorescent dye Coumarin-6 was encapsulated into the nanoparticles as a marker for the circulation studies. It was found that the blood florescence signal of NPs@HeLa decreased much slower than that of NPs. The AUC (0 -∞) and t1/2 of the NPs@HeLa were 2.7- and 2.2-fold higher than those of NPs, respectively (Figure 4E). Due to the “markers of self,” such as CD47 42, tumor cells can block the phagocytic function of macrophages. By mimicking this ability of tumor cells, HeLa cell membrane camouflaging enabled nanoparticles to escape from RES clearance.

### *In vivo* antitumor efficacy

**Figure 5:**
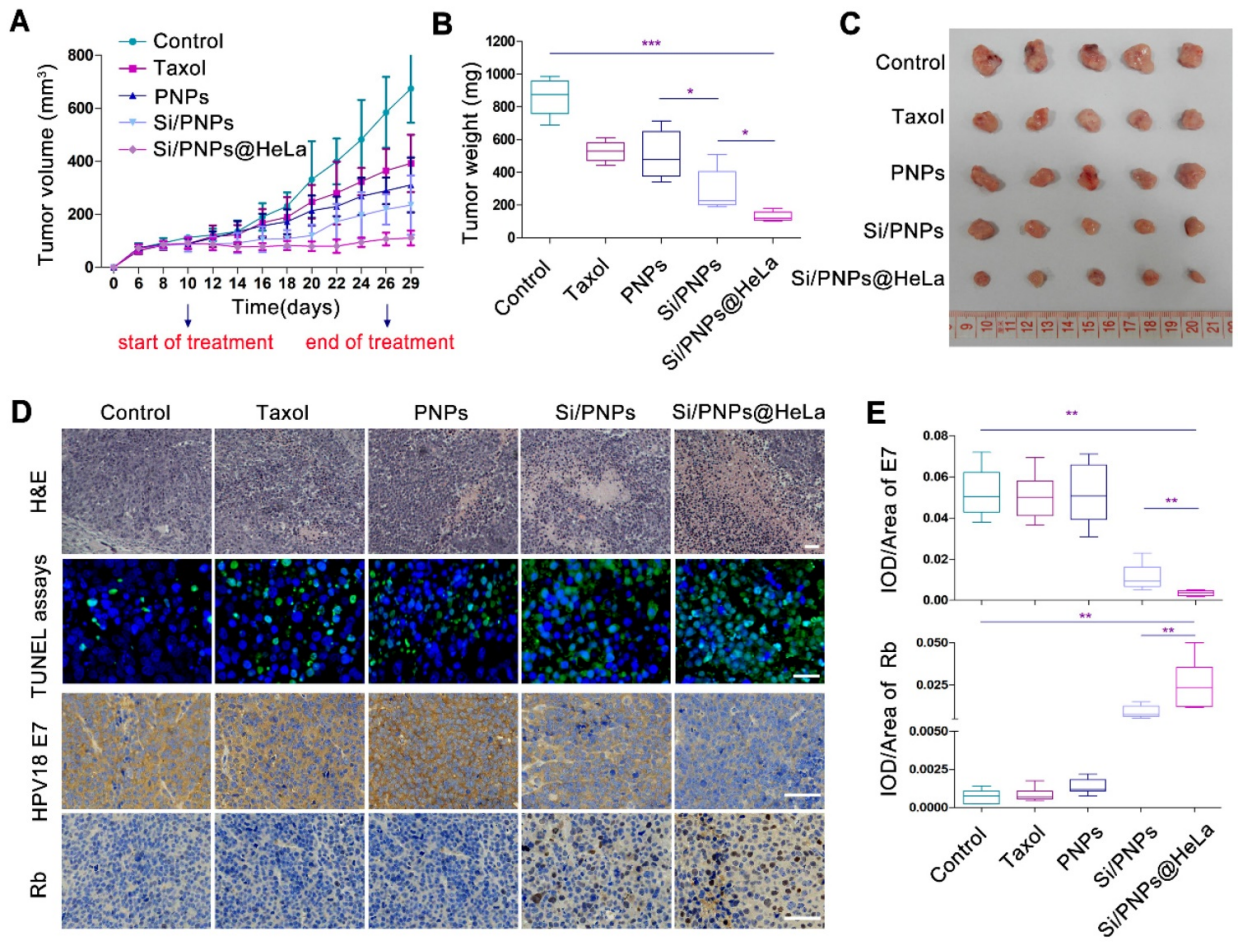
*In vivo* antitumor effects. (A) Tumor growth profiles of HeLa tumor-bearing mice receiving intravenous injections of different formulations every 2 days, for 9 injections, at a fixed PTX dose (6 mg/kg) and siRNA dose (2 mg/kg). Tumor weight (B) and photographs of the collected tumor tissues (C) on day 29. (D) Representative images of H&E staining (up, scale bar, 60 µm) and TUNEL assays (middle, scale bar, 25 µm) of tumor tissue; immunohistochemical analysis of the expression of HPV18 E7 and Rb (down, scale bar, 60 µm) in tumor sites. (E) Statistical data of HPV18 E7 and Rb expression using the optical staining intensities by ImagePro Plus (version 6.0). Data are given as the mean ± SD (n = 5 mice per group). *p < 0.05, **p < 0.01, ***p <0.001.

The subcutaneous HeLa xenograft model was used to investigate the impact of various PTX-based formulations on tumor growth *in vivo*. In order to confirm that an optimal anti-tumor effect could be achieved by our delivery system at a low dose, the total dose of PTX received by the mice in this study (6 mg/kg via a tail-vein injection, for a total of nine injections) was much lower than the human clinical equivalent dose 54. At this low dose, the tumor volume inhibiting rates of Taxol, PNPs, Si/PNPs and Si/PNPs@Hela were 41.85%, 53.92%, 65.14% and 83.6%, respectively. Taxol, the commercialized formulation of PTX, exhibited only a mild therapeutic effect. Similarly, because of the short half-life and poor specific targeting ability, a limited improvement in efficacy was observed in the PNPs group. By contrast, once co-delivered with siRNA-E7, a synergistic effect resulted in a significant amelioration in antitumor efficiency, which was further potentiated by camouflaging the NPs with the HeLa cell membrane. As a result, the smallest tumor volume (Figure 5A and C), the lowest tumor weight (Figure 5B), the largest necrotic area (assessed by H&E staining), and the most extensive tumor cell apoptosis (green) (Figure 5D) were observed in the Si/PNPs@HeLa group.

We further observed a down-regulation of HPV18 E7 and a restoration of Rb in tumor tissue in both Si/PNPs and Si/PNPs@HeLa groups using immunohistochemistry (IHC) staining. The gene silencing effect was significantly enhanced after HeLa cell membrane coating, possibly due to a better accumulation of nanoparticles in tumor tissues mediated by the tumor-homing ability of the HeLa-cell-biomimetic shell. As shown in Figure 5D and E, compared with the Si/PNPs group, the HPV18 E7 protein level in the Si/PNPs@HeLa group was much lower, while the restoration of downstream protein Rb was more pronounced. These data indicated that Si/PNPs@HeLa had a notable gene silencing ability, which is essential for achieving synergistic effects in combination therapy.

### *In vitro* and *in vivo* biosafety evaluation

**Figure 6:**
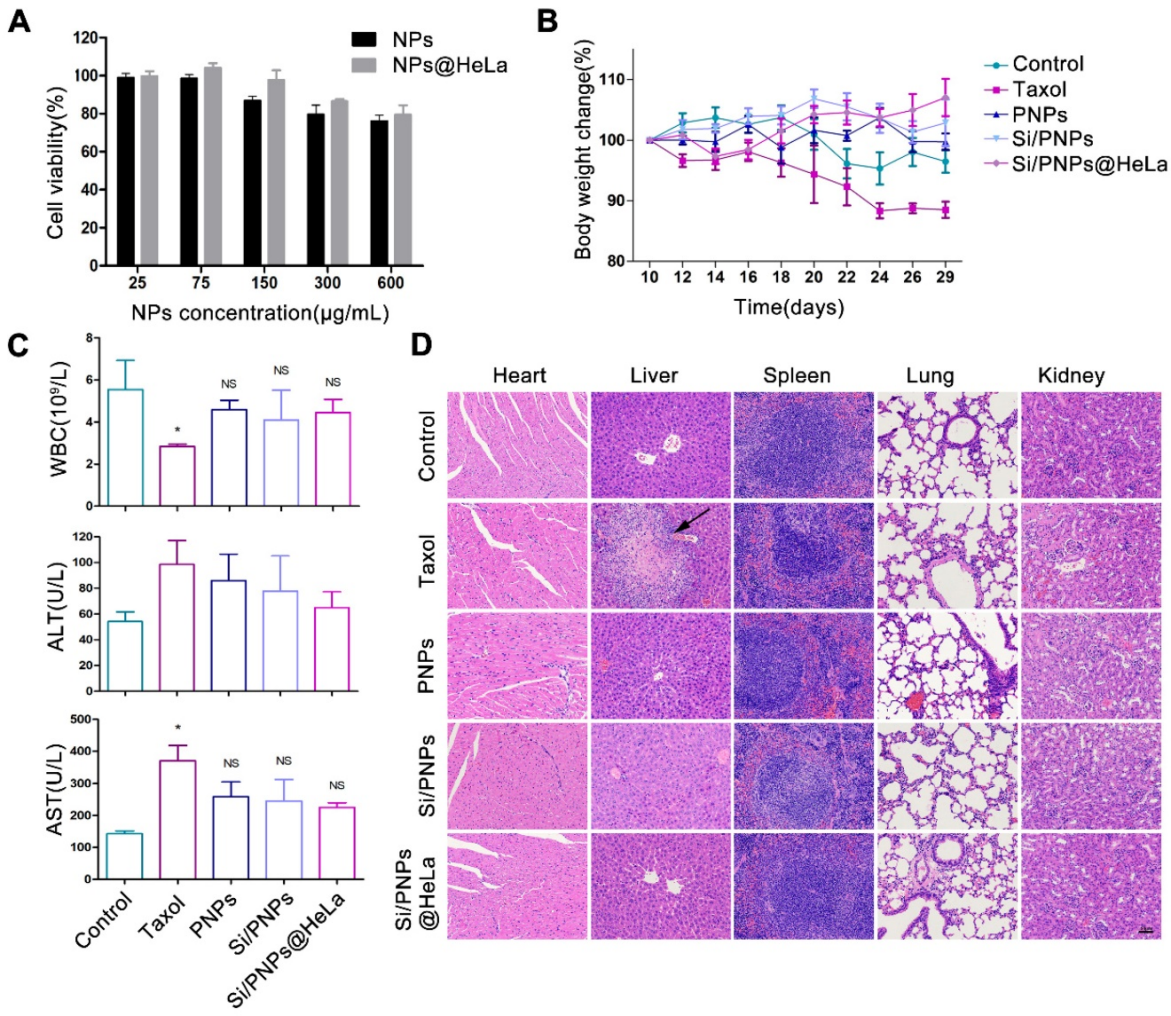
Biosafety estimation of various formulations. (A) Cytotoxicity of empty NPs and empty NPs@HeLa against HeLa cells after 72 h treatment with different concentrations. (B) Effects of different formulations on body weight of HeLa subcutaneous xenograft mice. (C) Test results of white blood cells count, plasma ALT, and AST at the end of treatment. (D) Representative H&E staining histological images of major organs from mice receiving different treatment. Scale bar, 60 µm. The arrow points to liver necrosis. Data are given as the mean ± SD (n = 5 mice per group). *p < 0.05, NS indicates *p* > 0.05.

Toxicity is a primary concern in the development of NP pharmaceuticals. Cytotoxicity was first evaluated in HeLa cells. The cell viability remained above 75% at all time points, even at concentrations of up to 600 µg/mL (Figure 6A and Figure S7), which indicated the low cytotoxicity of the nanocarriers. To further investigate the immunogenicity of NPs, various formulations were incubated with THP-1 human peripheral blood monocyte cells. The important inflammatory cytokines (IL-1β, IL-6, TNF-α) that reflect the immune response were measured by ELISA. As shown in Figure S8, no significant immunogenicity of NPs or free drugs was observed compared with the positive control.

*In vivo* biosafety assessment was performed in the HeLa tumor-bearing mice mentioned above, which were treated with PBS or various PTX-based formulations. Mice treated with PNPs, Si/PNPs, and Si/PNPs@HeLa showed negligible body weight changes throughout the whole treatment period, as opposed to the considerable weight loss observed in Taxol treated mice (Figure 6B). Since myelosuppression is a well-known adverse effect of PTX 55, the level of white blood cells (WBCs) was first measured. The mice treated with PNPs, Si/PNPs, or Si/PNPs@HeLa exhibited a less severe leukopenia compared to the mice receiving Taxol treatment (Figure 6C). Hepatotoxicity was further assessed as PTX is mainly metabolized by the liver. As shown in Figure 6C, serum levels of alanine aminotransferase (ALT) and aspartate aminotransferase (AST), two major indicators of hepatocellular injury, were markedly elevated in the Taxol treated mice. In contrast, ALT and AST levels of mice following treatment with PNPs, Si/PNPs, or Si/PNPs@HeLa were relatively lower. Histological assessments were accomplished at the endpoint of the study, and liver necrosis was clearly observed in the mice receiving Taxol treatment, whereas no pathological changes were observed in the slices of other groups (Figure 6D). All this information consistently demonstrated the good biocompatibility of Si/PNPs@HeLa and that encapsulation of PTX in the nanoparticles may alleviate the hematological toxicity and the hepatotoxicity of PTX to some extent.

## Conclusion

In summary, based on PLGA nanoparticles co-loaded with hydrophobic drugs and siRNA, a tumor-cell-like biomimetic nanoplatform was developed for precise combinational tumor therapy *in vivo*. In this nanosystem, HeLa cell membranes mediate tumor homing and immune escape to achieve highly specific targeted delivery of PTX and siRNA-E7 to the homologous tumor tissue. Moreover, while restoring the expression of the anti-tumor protein Rb, siRNA targeting E7 could overcome PTX-induced resistance by inhibiting the activation of the AKT pathway, and the resulting synergistic effect enhances tumor suppression in CC. It could be envisioned that this biomimetic nanoplatform can be further adapted for the co-delivery of other small molecule anticancer drugs and gene editing tools, such as miRNA and ASO, so as to exert a multimodal precise anticancer effect in a wide range of tumors. Meanwhile, this platform also provides the feasibility of developing personalized cancer therapies by disguising drug nanocarriers using tumor cell membranes derived from cancer patients.

## Supplementary Material

Supplementary figures and tables.

## Abbreviations

CC: cervical cancer
PTX: paclitaxel
PLGA: poly (lactic-co-glycolic acid)
PVA: Poly(vinyl alcohol)
PEI: polyethyleneimine
Si/PNPs@HeLa: HeLa cell membrane camouflaged siRNA/PTX co-loaded nanoparticles
DLS: dynamic light scattering
TEM: Transmission electron microscopy
CLSM: confocal laser scanning microscope
qRT-PCR: quantitative real-time PCR
ALT: aspartate aminotransferase
AST: alanine aminotransferase
ASO: antisense oligonucleotide
