# Supplementary material for: Bioinspired tumor-homing nanoplatform for co-delivery of paclitaxel and siRNA-E7 to HPV-related cervical malignancies for synergistic therapy
Source: Theranostics. 2020 Feb 10;10(7):3325–39. doi: 10.7150/thno.41228 (PMC7053183; doi:10.7150/thno.41228)
Supplement: Supplementary file 1 — Supplementary figures and tables. [file thnov10p3325s1.pdf]

Supporting Information

Bioinspired tumor-homing nanoplatform for co-delivery of paclitaxel and siRNA-E7 to HPV-related cervical malignancies for synergistic therapy

Cong Xu[1,2], Wan Liu[1,2], Yuan Hu[1,2], Weiping Li[1,2] and Wen Di[1,2]

1. Department of Obstetrics and Gynecology, Renji Hospital, School of Medicine, Shanghai Jiao Tong University, Shanghai 200127, People's Republic of China.
2. Shanghai Key Laboratory of Gynecologic Oncology, Renji Hospital, School of Medicine, Shanghai Jiao Tong University, Shanghai 200127, People's Republic of China.

Corresponding authors: liweiping@renji.com; diwen@renji.com

Table S1. Hydrodynamic size and Zeta potential of different samples measured by DLS.

samples	D_h (nm)[a]	PDI[b]	ζ Potential (mV)
NPs	169.4	0.079	-19.1
SiNPs	174.2	0.107	-18.7
PNPs	176.1	0.085	-12.9
Si/PNPs	170.9	0.091	-14.4
Si/PNPs@HeLa	194.1	0.143	-30
HeLa membrane-derived vesicles	379.6	0.190	-30.2

[a] The hydrodynamic size of nanoparticles were measured by DLS (n=3);
[b] Polydispersity index.

Table S2. Encapsulation efficiency and drug loading content of various nanoparticle formulations.

samples	EE [a] of PTX (%)	EE [a] of siRNA-E7 (%)	DL[b] of PTX (%)	DL[b] of siRNA-E7 (μg/10mg)
SiNPs	-	87.5 ± 1.69%	-	58.2 ± 1.12
PNPs	91.0 ± 2.26%	-	2.3 ± 0.05%	-
Si/PNPs	90.2 ± 0.43%	88.4 ± 0.22%	2.3 ± 0.10%	58.8 ± 0.15

[a] Encapsulation efficiency;
[b] Drug loading; Data are shown as mean ± SD (n=3).

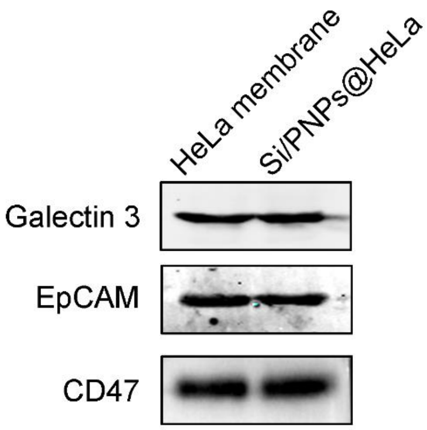

Figure S1. Western blot analysis of the Galectin-3, EpCAM, CD47 protein in HeLa membrane and Si/PNPs@HeLa lysates.

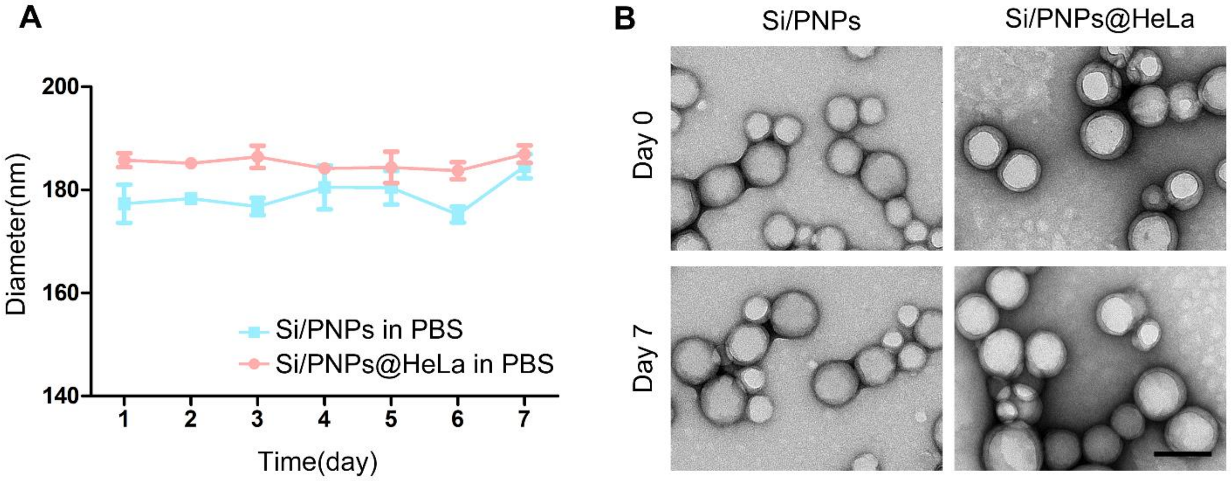

Figure S2. (A) Time-dependent size stability of Si/PNPs and Si/PNPs@HeLa in PBS at 37 °C. Data are shown as mean ± SD (n=3). (B) TEM images of Si/PNPs and Si/PNPs@HeLa in PBS at 37 °C on day 0 and day 7.

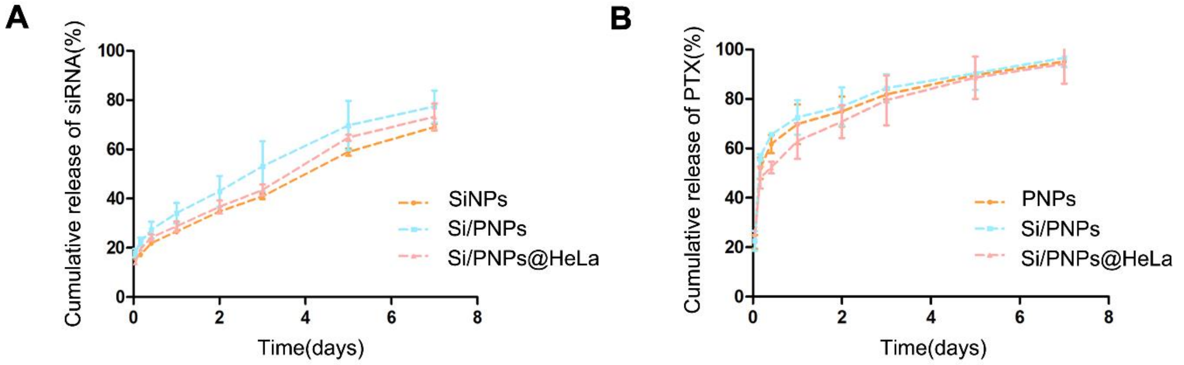

Figure S3. (A) In vitro release profile of siRNA from SiNPs, Si/PNPs and Si/PNPs@HeLa at 37 °C. (B) In vitro release profile of PTX from PNPs, Si/PNPs and Si/PNPs@HeLa at 37 °C. Data are shown as mean ± SD (n=3).

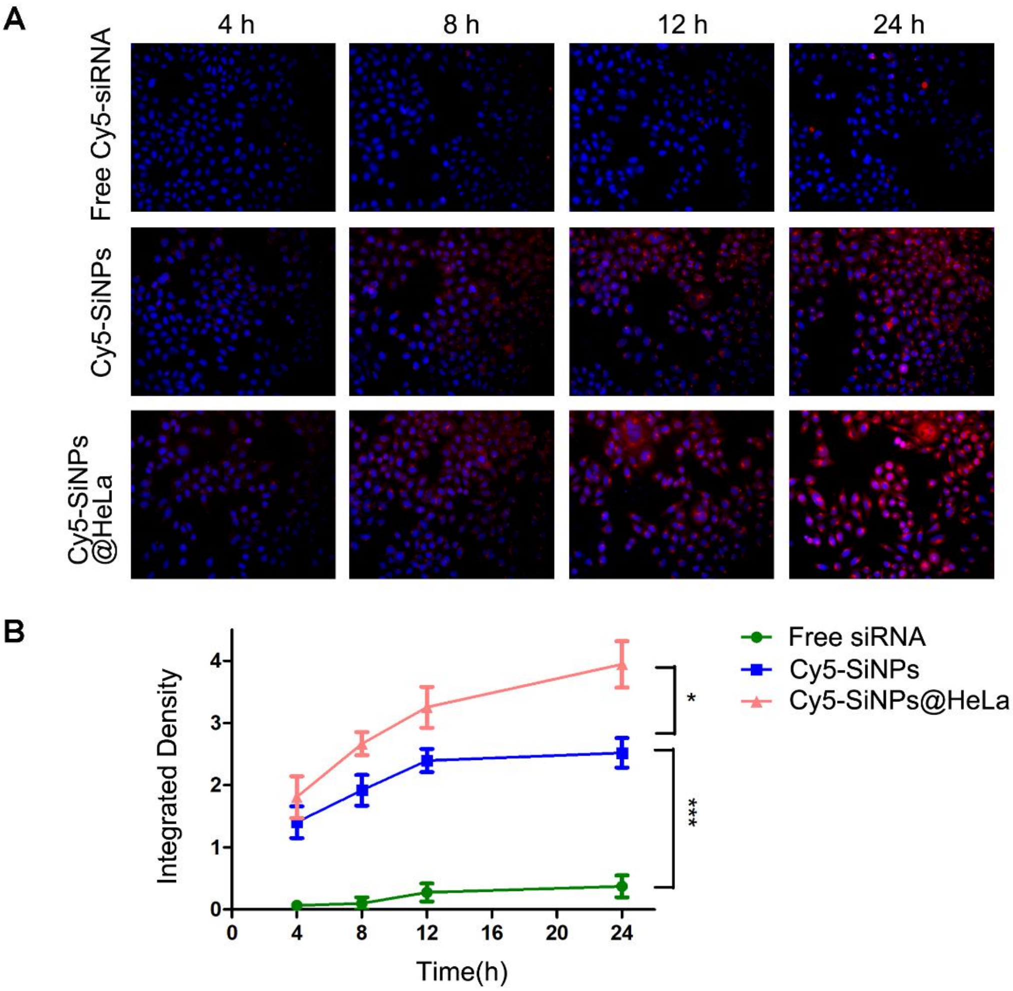

Figure S4. (A) Fluorescence microscopy images of HeLa cells after incubation with free Cy5 labeled-siRNA, Cy5 labeled SiNPs and Cy5 labeled SiNPs@HeLa (100 nM) for 4, 8, 12, and 24 h. (B) Quantification of intracellular Cy5 fluorescence intensity of (A) by Image J software. Data are shown as mean ± SD; $*p < 0.05$, $***p < 0.001$.

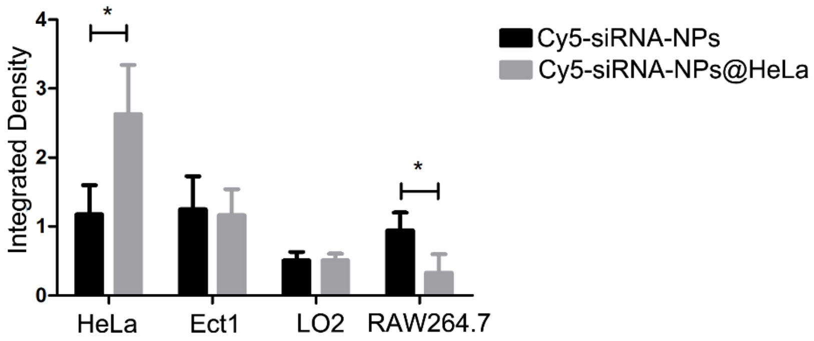

Figure S5. Intracellular Cy5 fluorescence intensity of HeLa, Ect1, LO2, RAW264.7 treated with Cy5 labeled SiNPs or SiNPs@HeLa for 3 h was quantified by Image J software. Data are given as mean ± SD; *$p < 0.05$.

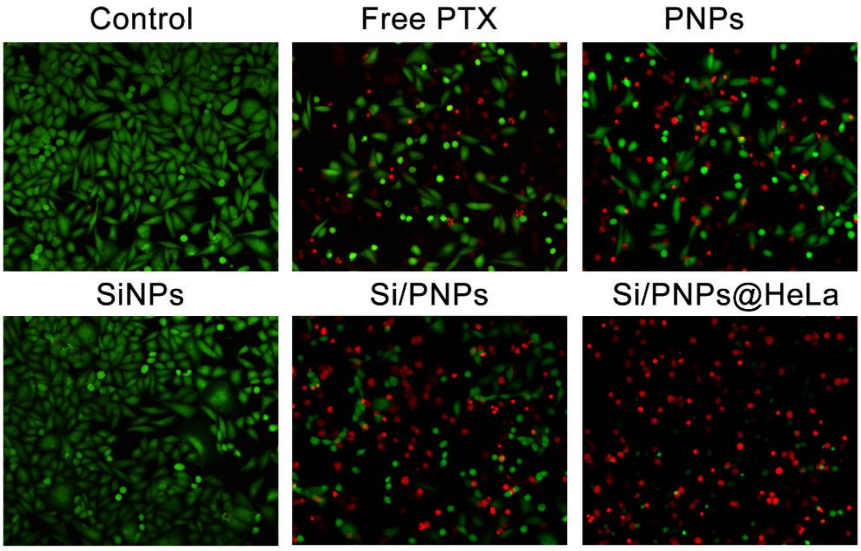

Figure S6. Calcein-AM/PI double stain kit was employed to detect HeLa cell death induced by free PTX, PNPs, SiNPs, Si/PNPs and Si/PNPs@HeLa at an equivalent PTX concentration of 10 nM and an siRNA concentration of 100 nM for 48 h.

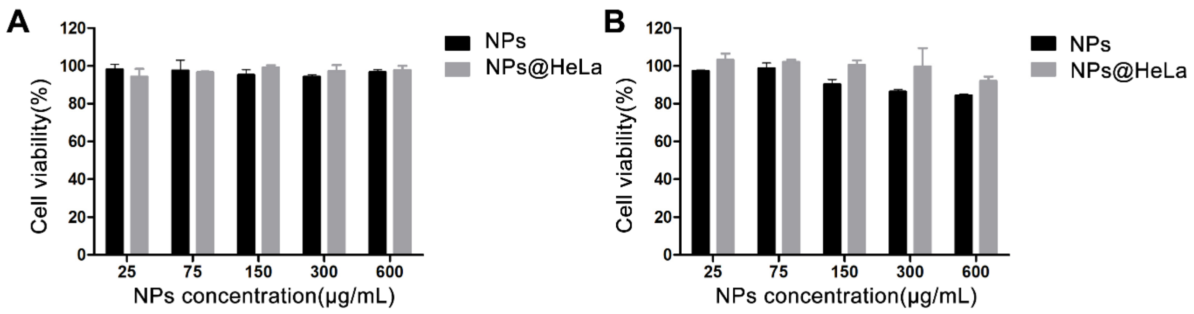

Figure S7. Cell viability of HeLa cells when incubated with empty NPs or NPs@HeLa at various concentrations for 24h (A), 48 h (B), respectively. Data are shown as mean ± SD (n=3).

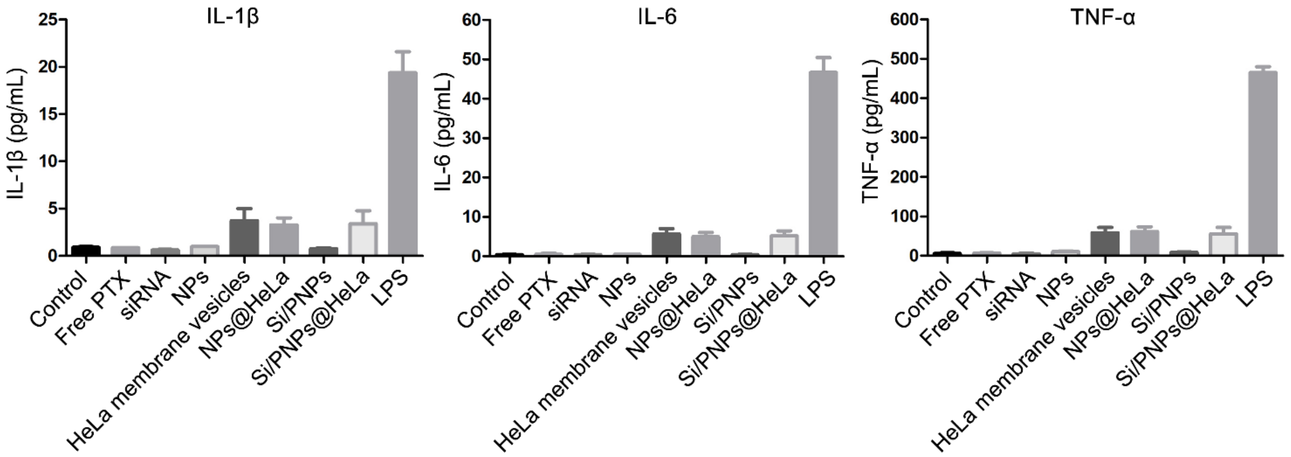

Figure S8. The immunogenicity of various formulations was evaluated by incubation with THP-1 cells for 24 h. The concentration of inflammatory cytokines (IL-1β, IL-6, TNF-α) in the cell supernatant were determined by ELISA. Lipopolysaccharides (3 μg/mL) were used as a positive control. Data are shown as mean ± SD (n=3).
